# Systematic transcriptome analysis reveals molecular mechanisms and indications of bupleuri radix

**DOI:** 10.3389/fphar.2022.1010520

**Published:** 2022-10-11

**Authors:** Sang-Min Park, Aeyung Kim, Haeseung Lee, Su-Jin Baek, No Soo Kim, Musun Park, Jin-Mu Yi, Seongwon Cha

**Affiliations:** ^1^ KM Data Division, Korea Institute of Oriental Medicine, Daejeon, South Korea; ^2^ College of Pharmacy, Chungnam National University, Daejeon, South Korea; ^3^ KM Application Center, Korea Institute of Oriental Medicine, Daegu, South Korea; ^4^ College of Pharmacy, Pusan National University, Busan, South Korea; ^5^ KM Convergence Research Division, Korea Institute of Oriental Medicine, Daejeon, South Korea

**Keywords:** herbal medicine, transcriptome, gene signature, pathway enrichment analysis, drug repurposing, wound healing

## Abstract

Pharmacogenomic analysis based on drug transcriptomic signatures is widely used to identify mechanisms of action and pharmacological indications. Despite accumulating reports on the efficacy of medicinal herbs, related transcriptome-level analyses are lacking. The aim of the present study was to elucidate the underlying molecular mechanisms of action of Bupleuri Radix (BR), a widely used herbal medicine, through a systematic transcriptomic analysis. We analyzed the drug-responsive transcriptome profiling of A549 lung cancer cell line after treating them with multiple doses of BR water (W-BR) and ethanol (E-BR) extracts and their phytochemicals. *In vitro* validation experiments were performed using both A549 and the immortalized human keratinocyte line HaCaT. Pathway enrichment analysis revealed the anti-cancer effects of BR treatment via inhibition of cell proliferation and induction of apoptosis. Enhanced cell adhesion and migration were observed with the W-BR but not with the E-BR. Comparison with a disease signature database validated an indication of the W-BR for skin disorders. Moreover, W-BR treatment showed the wound-healing effect in skin and lung cells. The main active ingredients of BR showed only the anti-cancer effect of the E-BR and not the wound healing effect of the W-BR, suggesting the need for research on minor ingredients of BR.

## Introduction

Pharmacological perturbations by drugs (synthetic chemicals or natural products) after binding to the drug targets activate cellular signal transduction and induce specific gene expression patterns (Lamb et al., 2006). Therefore, transcriptomic signatures are considered ideal for identifying the mode of action (MoA) and molecular mechanism of a drug (Chen and Butte, 2016). One of the large-scale public databases of transcriptomic signatures for drug perturbations is the Connectivity Map (CMap), supplied by the Library of Integrated Network-based Cellular Signatures program that provides genome-wide expression profiles of approximately 40,000 drugs across various cell lines (Subramanian et al., 2017). Based on pharmacogenomic databases, the approach of analyzing and comparing transcriptome signatures is widely used to unravel unknown mechanisms of action, discover novel indications, and repurpose drugs. For example, topiramate and cimetidine have been suggested as treatments for inflammatory bowel disease and lung adenocarcinoma, respectively, because their transcriptomic signatures are negatively correlated with differentially expressed genes associated with these diseases (Dudley et al., 2011; Sirota et al., 2011). Moreover, bortezomib and atorvastatin were identified as potent drugs to reverse the metastatic effect of lung cancer and its resistance to anti-cancer drugs, respectively, using the CMap database (Hong et al., 2018; Kwon et al., 2020).

As medicinal herbs contain many constituents, they have the advantage of eliciting a multi-functional effect on multiple disease targets (Li et al., 2022). However, a comprehensive characterization of the molecular mechanisms and downstream targets of herbal extracts and related phytochemicals is still lacking. In the drug-responsive transcriptome data of the CMap, phytochemicals account for less than 2% of the tested compounds (Subramanian et al., 2017). The largest transcriptome dataset of herbal medicine constituents provides information on 102 components used in the treatment of one cell line at a single dose (Lv et al., 2017). Nevertheless, the overall molecular effect of herbs may differ from the sum of effects of individual components of the herb owing to synergism or antagonism between the components. Additionally, even the same herb may have different medicinal effects when distinct extraction methods are used. Despite this, only a few transcriptome datasets are available for herbal extracts in HERB—a recently constructed database (Fang et al., 2021). Therefore, it is necessary to systematically generate and analyze transcriptome data for herbal medicines (Baek et al., 2022).

Bupleuri Radix (BR) is the dried root of *Bupleurum falcatum* Linne, *Bupleurum chinense* DC, and *Bupleurum scorzonerufolium* Wild. BR has been used as a traditional medicine for over 2000 years and is regarded as one of the most important herbal medicines in Asian countries, including China, Japan, and Korea (Yang et al., 2017). In recent pharmacological studies, BR has been demonstrated to possess beneficial effects, including anti-cancer, anti-pyretic, anti-inflammatory, anti-depressant, anti-viral, hepatoprotective, neuroprotective, and immunomodulating activities (Yang et al., 2017). Many bioactive ingredients have been isolated from BR, including triterpenoid saponins, polyacetylenes, flavonoids, lignans, fatty acids, and sterols. Among them, triterpenoid saponins, especially saikosaponin a (SSa) and saikosaponin d (SSd), are believed to be responsible for the broad spectrum of pharmacological activities of BR (Yuan et al., 2017). Contrary to the accumulation of efficacy studies revealed through pharmacological experiments, the investigation of transcriptome signatures by genome-wide analysis for BR treatment remains to be conducted.

Here, we generated the drug-responsive transcriptome data for BR extracts and their major components, which were tested on the A549 lung cancer cell line with multiple doses and extraction methods. Systematic transcriptome analysis revealed the molecular pathways associated with the mechanism of BR and highlighted the differences between the effects of water and ethanol extracts of BR. To identify indications of BR, we also compared transcriptome profiling of BR extracts with disease-related signatures. Our study demonstrated that a comprehensive analysis using the drug-responsive transcriptome data can provide insights into the molecular mechanisms as well as indications of herbal medicines.

## Materials and methods

### Preparation of W-BR, E-BR, and saikosaponins

Lyophilized powders of water (W-BR; 3-19-0054) and 70% ethanol (E-BR; 3-19-0097) extracts of BR were obtained from the Herbal Medicine Resources Research Center, Korea Institute of Oriental Medicine (Naju, Republic of Korea). In brief, dried and crushed BR (1 kg) was extracted with water under reflux for 3 h (W-BR) or 70% EtOH under sonication for 1 h and repeated two times (E-BR). After extraction, the extract solutions were filtered, evaporated, freeze-dried, and homogenized using a 600 µm sieve. The extraction yields were 20.26% (W-BR), and 9.46% (E-BR) respectively. Each compound was extensively dissolved in 2% dimethyl sulfoxide (DMSO) (Sigma-Aldrich Co., St Louis, MO, United States) to obtain a final concentration of 10 mg/ml by vigorous vortexing, filtered through a 0.22 µm membrane (Sartorius Stedim Biotech GmbH, Goettingen, Germany), and stored at −20°C until use. The preliminary quantitative analysis of E-BR and W-BR confirmed that saikosaponins were major compounds, and the two extraction methods produced extracts that differed in composition (Choi et al., 2021). Saikosaponins, including SSa, saikosaponin b (SSb) 1–4, saikosaponin c (SSc), and SSd, were purchased from the Natural Product Institute of Science and Technology (Anseong, Republic of Korea) and dissolved in DMSO to obtain a 20 mM concentration. The purity of all saikosaponins was greater than 99.9%, as determined by high-performance liquid chromatography.

### Cell culture

The human lung adenocarcinoma A549 cell line (CCL-185) was obtained from the American Type Culture Collection (Manassas, VA, United States) and cultured in RPMI-1640 supplemented with 10% heat-inactivated fetal bovine serum, 100 IU/ml penicillin, and 100 μg/ml streptomycin. The human keratinocyte HaCaT cell line was obtained from CLS Cell Line Service GmbH (Eppelheim, Germany) and cultured in DMEM supplemented with 10% fetal bovine serum, 100 IU/ml penicillin, and 100 μg/ml streptomycin. The cells were maintained in a humidified 5% CO_2_ incubator at 37°C. All basal media and supplements for cell culture were obtained from Thermo Fisher Scientific (Rockford, IL, United States).

### Drug treatment and total RNA preparation for RNA sequencing analysis

To determine the treatment drug concentrations, we performed cell cytotoxicity teststo investigate drug doses exhibiting 80% cell viability (IC20s), and the determined IC20s were selected as the maximum doses for obtaining RNA-seq data. For drugs whose IC20 could not be determined, the highest treatment concentrations were set to 500 μg/ml for extracts and 10 μM for phytochemicals, considering the solubility and clinical application of the drugs. To confirm the influence of concentration, we treated the cells with three concentrations in 1/5 serial dilutions. Consequently, the cells were exposed to three doses (low, medium, high) of W-BR (20, 100, and 500 μg/ml), E-BR (5.32, 26.6, and 133 μg/ml), saikosaponins (SSa, SSb1–4, SSc at 0.4, 2, and 10 μM; SSd at 0.2, 1, and 5 µM), or combinations of seven saikosaponins (0.08, 0.4, and 2 µM of each). Wortmannin (1.95 µM), trichostatin A (0.64 µM), and vorinostat (10 µM) were also included in the assay plates as positive control drugs for comparison with the CMap data. DMSO (0.1%) was used as the vehicle control. One day before drug treatment, A549 cells were plated in a 6-well culture plate (3 × 10^5^ cells/well) containing 3 ml of growth medium. After 24 h of drug treatment, the cells were washed three times with ice-cold phosphate-buffered saline, and total RNA was isolated using TRIzol RNA Isolation Reagents (Thermo Fisher Scientific) according to the manufacturer’s instructions. The concentration of isolated RNA was determined using an Agilent RNA 6000 Nano kit (Agilent Technologies, Waldbronn, Germany), and RNA quality was evaluated by assessing the RNA integrity number (RIN >7).

### Acquisition and pre-processing of RNA-seq data

Total RNA (1 µg) was processed to prepare the mRNA sequencing library using the MGIEasy RNA Directional Library Prep kit (MGI Tech Co., Ltd., China) according to the manufacturer’s instructions. The library was quantified using the QauntiFluor^®^ ssDNA System (Promega Corporation, WI, United States). The prepared DNA nanoball was sequenced on the MGIseq system (MGI Tech Co., Ltd., China) with 100 bp paired-end reads. RNA-seq raw data quality was checked using FastQC (v0.11.9). TrimGalore (v0.6.5) was used to remove the common regions of the MGISEQ adapter sequences. The cleaned reads were mapped to the human genome assembly GRCh38 (hg38) using the STAR aligner (v2.7.3a) with default settings. Transcript abundance per gene, such as expected read count or transcripts per million, was quantified by RSEM (v1.3.3) with human gene annotation GRCh38.84. The raw sequence data (FASTQ files) and pre-processed count data were deposited in the Gene Expression Omnibus with accession number GSE182007. Differential gene expression analysis between the groups (e.g., BR treatment vs. vehicle) was conducted using the edgeR package (v3.38) in R (v3.6.3), yielding a ranked list of genes based on log_2_FC and adjusted *p*-value.

### Gene set enrichment analysis

Given a ranked list of genes based on differential expression, GSEA was performed for the well-curated gene sets (hallmark, Gene Ontology biological process, and canonical pathways integrating BioCarta, KEGG, PID, REACTOME, and WikiPathways) in the Molecular Signature Database (MSigDB v7.4.1). The GSEA method quantifies the degree to which genes in a gene set are overrepresented at the top or bottom of the ranked list of genes. GSEA was performed using the fgsea package (v1.20) in R (v4.0.3) using parameters of minimum size 15, maximum size 500, and 10,000 permutations. The statistical significance of all GSEA results were evaluated by adjusted *p*-value using multiple testing corrections *via* false discovery rate (FDR) estimation. A network analysis using the GSEA results was performed with the EnrichmentMap application (Merico et al., 2010) of Cytoscape (v3.8.0).

### Connectivity map data analysis to infer MoAs and pharmacological indications of bupleuri radix

We downloaded CMap data from the clue.io platform (clue.io/data/CMap2020#LINCS2020) and leveraged gene expression signatures (level 5: moderated z-scores) from the treatment of A549 cell lines with 384 launched drugs. From the Clue Repurposing Hub (clue.io/repurposing-app), we retrieved information on clinical phases, 190 MoAs, 602 targets, and 22 indications of the 384 drugs. Signatures with poor reproducibility (distil_cc_q75’ < 0.2 and pct_self_rank_q25 > 0.05) were filtered out. The R package cmapR (v1.8.0) was used to access and manipulate a level 5 GCTX file (level5_beta_trt_cp_ n720216 × 12,328. gctx). To measure the similarity of the expression signatures of BR and the CMap drugs, we converted gene-level expression to pathway-level (Supplementary Figure S1). Gene expression changes induced by BR or CMap drugs were quantified using two different platforms, RNA-seq and L1000 assays, respectively. Thus distributions of expression values from the two platforms were quite distinct, making the direct comparison between gene expression values difficult. As an alternative to comparing expression signatures obtained from two different platforms, we took advantage of GSEA to reveal the genome-wide perturbing effects of BR and CMap drugs at the pathway level. We utilized well-curated 6,482 pathway gene sets from MsigDB (C2) and performed a series of GSEA on the expression signatures of BR and the CMap drugs. From the results of GSEA, we defined pathway activity score (PAC) as a ‘sign (enrichment score) × -log_10_(*p*-value)’ value to indicate the degree of significance. PAC vectors of equal length (*n* = 6,482) were generated for BR and all CMap drugs. Subsequently, we calculated the Pearson correlation coefficient between PAC vectors of BR and each CMap drug. Based on the similarity of BR and 384 CMap drugs, we could infer MoA or indication of BR from known MoAs or indications of the drugs most similar to BR. We first collected the known associations between 384 drugs and 190 MoAs or 22 indications. We then conducted a one-sided Fisher’s exact test to quantify whether drugs corresponding to each of 190 MoAs or 22 indications had high coefficients. Finally, each MoA or indication was given a single enrichment score, -log_10_(*p*-value), indicating the degree of over-representation of the corresponding drugs within the top 10% based on the correlation coefficient.

### Prediction of indication of W-BR

The CREEDS database contains upregulated and downregulated differentially expressed genes for 324 human diseases (Wang et al., 2018). To focus on the therapeutic effects of W-BR on functional dysregulation, we first identified altered pathways of each disease via an over-representation analysis using the hypergeometric test with 6,482 pathways from the well-curated gene sets. After FDR-based multiple testing correction, significantly enriched pathways for upregulated or downregulated differentially expressed genes of each disease with a Q-value < 0.05 were selected and termed as up- or down-pathways of the disease. Next, up- and down-pathways of diseases were compared one-to-one to down- and up-pathways of W-BR, respectively, and the number of common pathways obtained from both comparisons were summed.

### Cell cytotoxicity assay

To evaluate the effect of W-BR, E-BR, SSa, and SSd on cell proliferation, we performed cells seeding on a 96-well culture plate at a density of 5 × 10^3^/well/100 μL and treated them with the indicated concentrations of W-BR, E-BR, SSa, and SSd for 24 h. The relative cell viabilities were measured using the EZ-Cytox Enhanced Cell Viability Assay kit (Daeil Lab Service Co., Ltd., Seoul, Republic of Korea) according to the manufacturer’s instructions. Colorimetric absorbance was determined at 450 nm using a SpectraMax3 microplate reader (Molecular Devices, LLC, Sunnyvale, CA, United States).

### Immunoblotting

Whole-cell lysates were prepared using a mammalian protein extraction reagent (Thermo Fisher Scientific), and protein concentrations were determined using a bicinchoninic acid kit (Thermo Fisher Scientific). Total protein (25 μg) in each cell lysate was resolved by sodium dodecyl sulfate-polyacrylamide gel electrophoresis and immunoblotted using specific antibodies. Proteins were visualized using the Clarity™ Western ECL substrate (Bio-Rad, Hercules, CA, United States) and ImageQuant™ LAS 4000 mini (GE Healthcare, Piscataway, NJ, United States). Band intensities were measured using the ImageJ software (v1.53), and the relative expression value of each protein was calculated after normalization based on β-actin expression level. Antibodies against α-actinin (#6487), focal adhesion kinase (FAK) (#3285), talin-1 (#4021), and vinculin (#4650) were obtained from Cell Signaling Technology, Inc. (Danvers, MA, United States), and anti-β-actin antibodies were obtained from Santa Cruz Biotechnology, Inc. (Santa Cruz, CA, United States).

### Cell-to-extracellular matrix adhesion assay

A549 cells pretreated with W-BR, E-BR, SSa, and SSd for 24 h were harvested using TrypLE™ Express solution (Thermo Fisher Scientific) and resuspended in a serum-free medium at 2 ×10^5^ cells/mL. Cells (2 × 10^4^ cells/well) were added to the wells of collagen I, collagen IV, or poly-d-lysine/laminin-coated 96-well culture plates (Corning Life Sciences, Kennebunk, ME, United States). After incubation for 30 min, the cells were washed three times with phosphate-buffered saline to remove unbound floating cells, and the attached cells were stained with a crystal violet solution (0.2% crystal violet in 20% methanol) for 30 min at room temperature. After washing with distilled water, stained cells were dissolved in 200 μl of 1% sodium dodecyl sulfate solution, and the absorbance at 590 nm was measured using a SpectraMax3 microplate reader (Molecular Devices, LLC).

### Migration assay

For the transwell cell migration assay, W-BR, E-BR, SSa, and SSd-treated or untreated A549 cells (1 × 10^5^/well) were resuspended in 100 μl of serum-free culture medium and added onto the upper side of the cell culture insert with an 8 μm pore size PET membrane (BD Falcon, Franklin Lakes, NJ, United States). On the lower side of 24-well culture plates, 600 μl of culture medium containing 10% fetal bovine serum was added to induce cellular chemotaxis. After incubation for 24 h, the cells on the upper surface of the insert were removed with a cotton swab. Cells that migrated to the lower surface of the insert were stained with a crystal violet solution and counted under a phase-contrast microscope. To measure the ability to migrate to the wounded area, we treated A549 cells grown to 90% confluence in a 12-well culture plate with 25 μg/ml mitomycin C (Sigma-Aldrich Co.) for 30 min and wounded them by scraping. After washing out the floating cell debris completely, we incubated the cells in a culture medium with or without W-BR, E-BR, SSa, and SSd for 48 h, and cell migration was monitored under a phase-contrast microscope. The wound closure ability of HaCaT cells pretreated with or without W-BR and E-BR was determined using the Radius™ 96-well Cell Migration Assay kit (Cell Biolabs Inc., San Diego, CA, United States) according to the manufacturer’s instructions.

### Statistical analysis

Data are presented as mean ± standard deviation, and statistical significance was analyzed using GraphPad Prism (GraphPad LLC, San Diego, CA, United States). Comparisons of means between two groups or among more than three groups were analyzed using two-tailed Student’s *t*-test and one-way analysis of variance, respectively. Differences were considered statistically significant at *p* < 0.05.

## Results

### Transcriptomic signatures of bupleuri radix extracts are associated with anti-cancer effects

**FIGURE 1:**
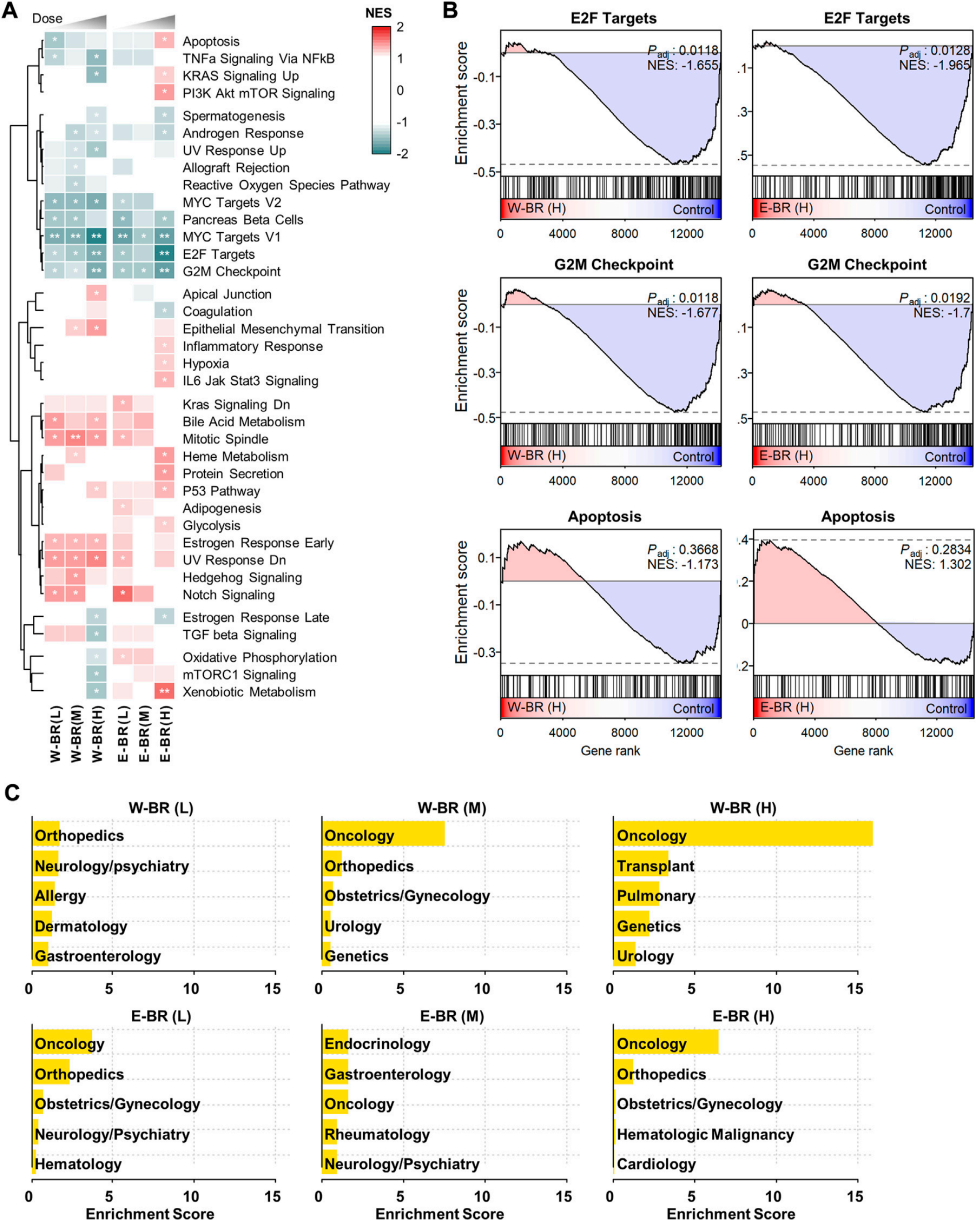
Transcriptomic signatures of bupleuri radix water extract (W-BR) and bupleuri radix ethanol extract (E-BR). **(A)** Dose-dependent gene set enrichment analysis results of W-BR and E-BR with the 50 hallmark gene sets. Only 37 gene sets with at least one condition showing a change of adjusted *p*-value (*P*_adj_) < 0.3 were displayed. * *P*_adj_ < 0.3 and ** *P*_adj_ < 0.05 vs. DMSO vehicle control. **(B)** The selections related to anti-cancer effects of W-BR (left) and E-BR (right). **(C)** The top-enriched indications of drugs whose expression signatures were similar to those of W-BR (upper) and E-BR (lower). A total of 384 FDA-approved drugs in the Connectivity Map database were used for comparison. The enrichment score is defined as–log_10_ (*p*-value) estimated in the hypergeometric test. L, M, and H denote the conditions of low, medium, and high dose, respectively. E-BR: Bupleuri radix ethanol extract; W-BR: Bupleuri radix water extract.

To investigate the transcriptomic signatures of BR extracts, we generated RNA-seq data from A549 cells, one of the core CMap cell panel cell lines, after treatment with either W-BR or E-BR at three increasing doses. After obtaining differentially expressed genes compared to the vehicle treatment, we performed GSEA using the hallmark gene sets representing well-defined biological processes with coherent expression. The resulting enrichment patterns were largely similar between W-BR and E-BR, and the normalized enrichment scores usually increased or decreased gradually, consistent with the drug dose (Figure 1A). Specifically, the pathways associated with anti-cancer effects were altered by treatment with W-BR and E-BR (Figure 1B). Moreover, pathways associated with the cell cycle and proliferation, including E2F targets, G2M checkpoint, and MYC targets were decreased following treatment with W-BR or E-BR. The apoptotic pathway was increased by E-BR but not by W-BR. Additionally, hypoxia and the inflammatory response were increased by E-BR, whereas apical junction and epithelial-mesenchymal transition were more significantly increased following treatment with W-BR.

Subsequently, we investigated MoAs and indications of known drugs that induced gene expression changes similar to those induced by BR in the CMap database. Changes in gene expression (collectively termed gene signatures) induced by BR treatment were compared with all the gene signatures of 384 FDA-approved drugs in the CMap database (Supplementary Figure S1A). Drugs that induce highly similar expression signatures are postulated to have similar physiological effects on cells. Most of the drugs highly similar to BR were enriched with anti-cancer drugs (Figure 1C), and their known MoAs included the inhibition of epidermal growth factor receptor, RAF, MAPK/ERK Kinase, SRC, and mTOR signaling pathways (Supplementary Figure S2A).

To assess the reproducibility and reliability of transcriptome data obtained from our in-house culture and treatment system, we generated RNA-seq data using three drugs designated as positive control drugs in the CMap database, vorinostat, trichostatin A, and wortmannin, which induce strong and consistent changes in gene expression. The original indications and MoAs of the three drugs were accurately predicted (Supplementary Figures S2B, S2C). Furthermore, we compared the GSEA results from our RNA-seq data and CMap data and showed similar perturbational patterns across various biological functions (Supplementary Figure S3). Taken together, these results support that drug-induced transcriptome data can be utilized to identify pharmacological indications and MoAs of a single or combinations of multiple complex compounds.

### Adhesion and migration pathways are enhanced in W-BR compared with E-BR

**FIGURE 2:**
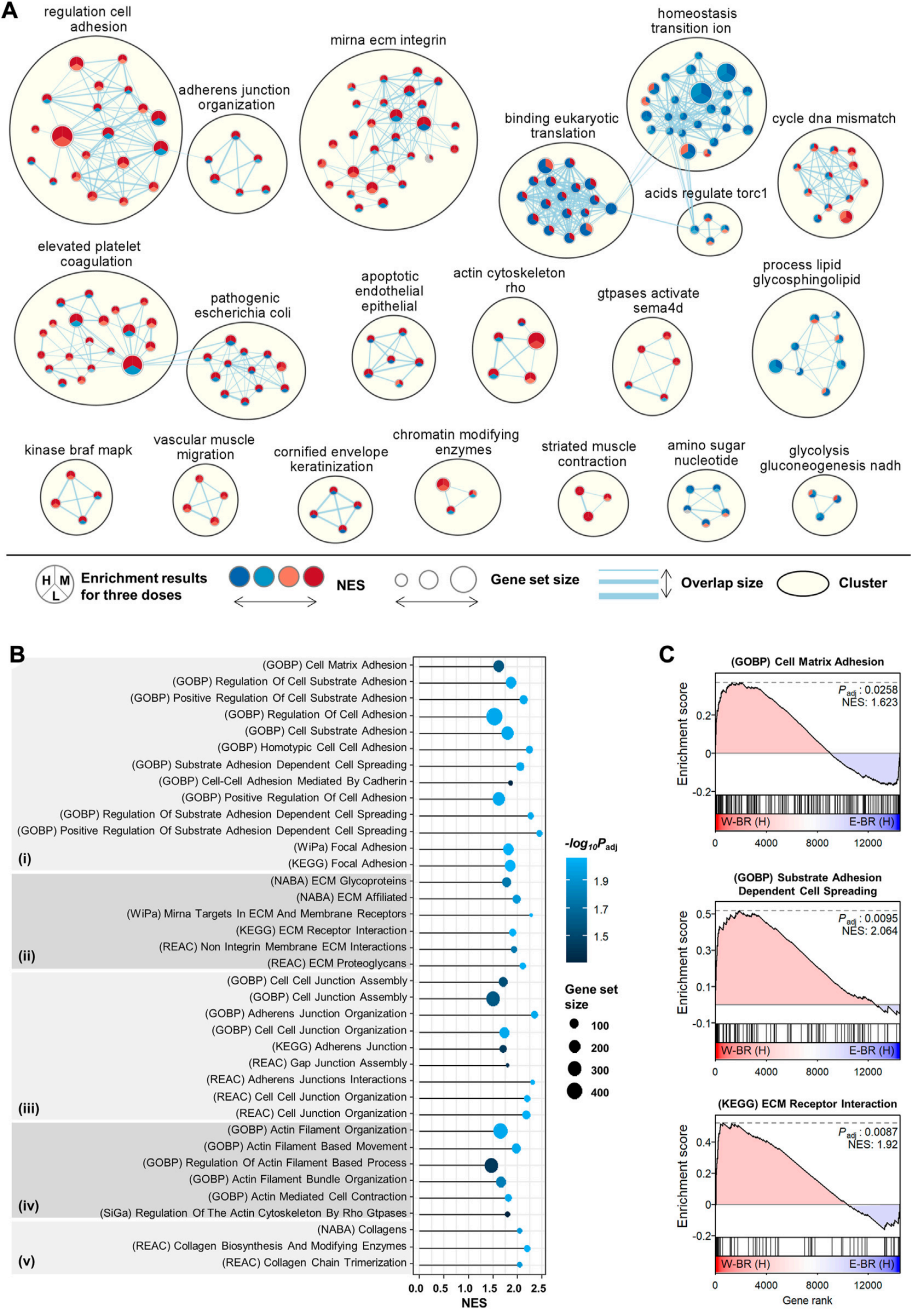
Enriched pathways in bupleuri radix water extract (W-BR) compared to bupleuri radix ethanol extract (E-BR). **(A)** Network analysis of enriched pathways using the EnrichmentMap application in Cytoscape. The curated and ontology gene sets were included from the misigDB. The clusters with more than three pathways were represented. **(B)** gene set enrichment analysis (GSEA) results for the selected pathways involved in adhesion **(i)**, extracellular matrix **(ii)**, junction **(iii)**, actin **(iv)**, and collagen **(v)**. **(C)** The selected GSEA plots for cell-matrix adhesion, cell-substrate adhesion, and cell spreading. L, M, or H denotes the conditions of low, medium, or high dose, respectively. GOBP, Gene Ontology Biological Processes; WiPa, Wikipathway; REAC, Reactome; KEGG, Kyoto Encyclopedia of Genes and Genomes; NABA, Naba **(A)** E-BR: bupleuri radix ethanol **extract**; W-BR: bupleuri radix water extract.

The obatined GSEA profiles showed different effects as well as common effects between W-BR and E-BR compared to controls (Figure 1). To highliht the distinct transcriptomic effects of W-BR and E-BR, we performed GSEA directly for the differential expression of W-BR vs. E-BR for each of the three doses using 10,403 pathways from the curated and ontology gene sets. Network analysis of the GSEA results using EnrichmentMap showed 20 functional clusters for enriched pathways (Figure 2A). Among the enriched clusters for W-BR, cell adhesion, adherens junction, and ECM were the largest clusters. We also noted that the cluster of the actin cytoskeleton was enriched with W-BR treatment. The clusters of ion transition and eukaryotic translation were enriched with E-BR treatment but discrepant according to dose.

We focused on the newly identified effects of W-BR by searching pathways with the keywords “adhesion,” “ECM,” “junction,” “actin,” and “collagen.” All searched significant pathways (adjusted *p* < 0.05) were higher with W-BR than with E-BR (Figure 2B). The pathways of cell-matrix adhesion, substrate adhesion-dependent cell spreading, and focal adhesion were specifically increased by W-BR (Figure 2C). Additionally, the pathways of focal adhesion, cell-cell junction assembly, actin-mediated cell contraction, actin filament-based movement, collagen biosynthesis, and modifying enzymes were increased. These results suggest that W-BR has different adhesion and migration effects than E-BR.

### W-BR increases and E-BR decreases focal adhesion protein levels

**FIGURE 3:**
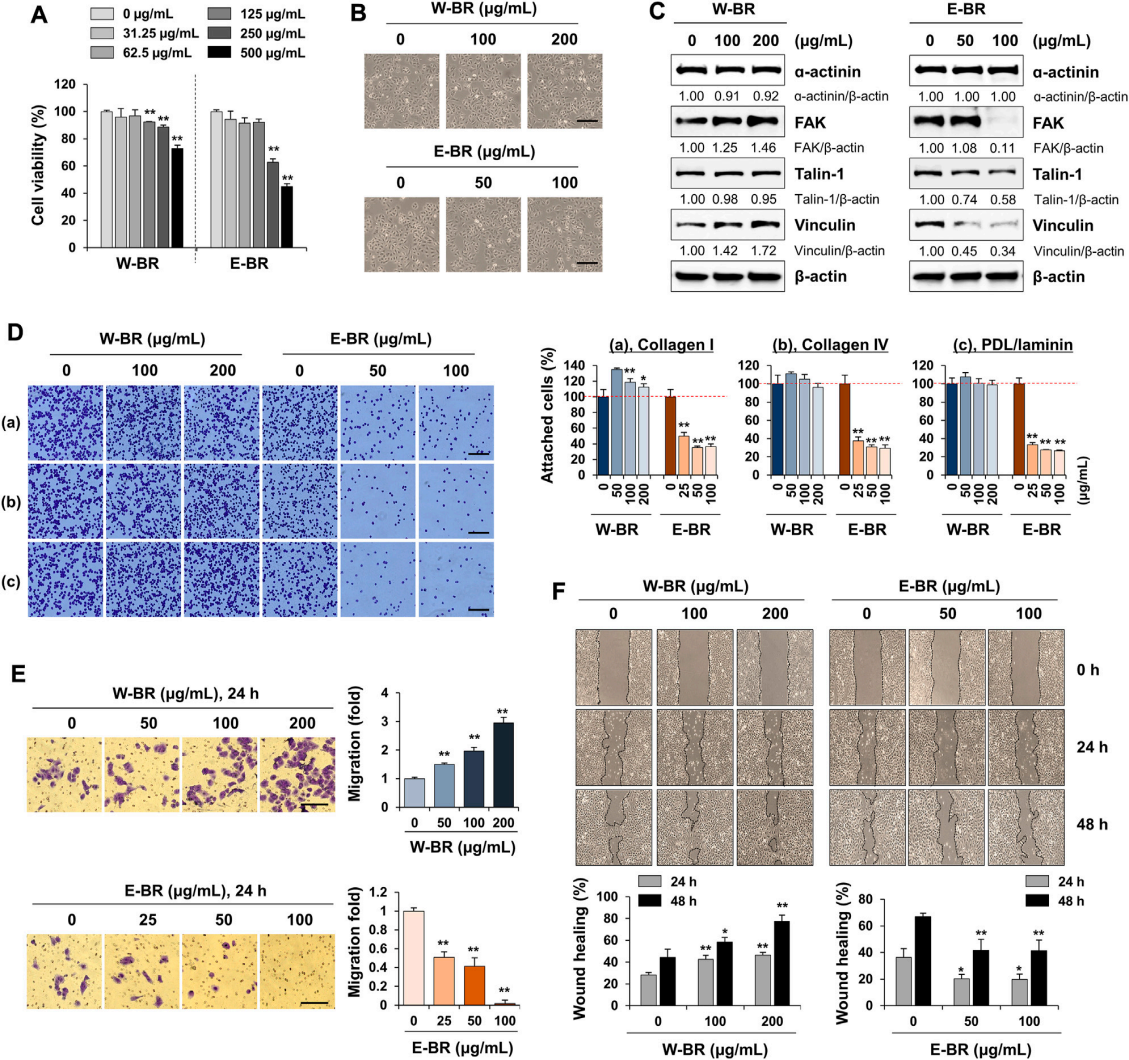
Effects of bupleuri radix water extract (W-BR) and bupleuri radix ethanol extract (E-BR) on proliferation, adhesion, and migration of A549 cells. **(A)** A549 cells were treated with W-BR or E-BR for 24 h, and viable cells were counted using the EZ-Cytox reagent. Relative cell viability compared with vehicle-treated cells is expressed as means ± SD. **(B)** A549 cells seeded on 24-well culture plates were treated with W-BR and E-BR for 24 h. Cells were photographed under an inverted microscope. Scale bar = 100 μm. Original magnification × 100. **(C)** Expression of focal adhesion-related proteins was determined using immunoblotting in W-BR- and E-BR-treated A549 cells. β-Actin was used as loading control. After measuring protein band intensities using the ImageJ software, relative values compared with those for vehicle-treated control cells were calculated upon normalization to β-actin expression. Data are representative of two independent experiments. **(D)** A549 cells pretreated with or without W-BR and E-BR for 24 h were seeded onto collagen I, collagen IV, or poly-D-Lysine/laminin-coated wells and incubated for 30 min. After washing, attached cells were counted. Scale bar = 100 μm. Original magnification × 100. **(E)** A549 cells pretreated with or without W-BR and E-BR for 24 h were loaded on the upper side of the transwell chamber. After filling the lower side with 10% fetal bovine serum/RPMI, the cells were further incubated for 24 h. Migrated cells were stained using a crystal violet solution and then observed under a phase-contrast inverted microscope. Scale bar = 200 μm. Original magnification × 100. **(F)** A549 cells grown up to 90% confluence were treated with mitomycin C for 30 min, and then injury lines were made. After being washed, the cells were incubated in 10% fetal bovine serum/RPMI containing 100 and 200 μg/mL W-BR or E-BR for 48 h. Cell migration was monitored under a phase-contrast inverted microscope, and images were acquired at the indicated timepoints. The relative degrees of adhesion to extracellular matrix, Transwell migration and wound healing were evaluated using ImageJ, and data are expressed as mean ± standard deviation of triplicate samples. *p < 0.05 and **p < 0.01 vs. vehicle-treated control. E-BR: bupleuri radix ethanol **extract**; W-BR: bupleuri radix water extract, FAK: focal adhesion kinase; PDL: poly-D-Lysine.

To experimentally validate the transcriptome analyses, we first determined the cytotoxic effects of W-BR and E-BR on A549 cells after incubation at the indicated concentrations for 24 h. W-BR and E-BR were dissolved in 2% DMSO to a final concentration of 10 mg/ml, and 0.1% DMSO was used as the vehicle control. At concentrations up to 200 μg/ml W-BR and 100 μg/ml E-BR, cell viability was maintained over 90% with no significant change in cell morphology compared with that of untreated control cells (Figures 3A,B). Therefore, in subsequent studies, cells were treated with W-BR at 100 and 200 μg/ml and E-BR at 50 and 100 μg/ml. As W-BR and E-BR were predicted to have different effects on adhesion and migration, we next examined their effects on the expression of focal adhesion-associated proteins in A549 cells by western blotting. W-BR significantly increased the levels of FAK and vinculin in a dose-dependent manner but did not affect the levels of ɑ-actinin and talin-1. In contrast, E-BR reduced the levels of FAK, talin-1, and vinculin in a dose-dependent manner (Figure 3C).

### E-BR reduces adhesion of cell-to-extracellular matrix and migration potential of A549 cells

FAK is overexpressed in different cancer cells and plays a crucial role in maintaining malignancy, including adhesion, migration, and proliferation (Tai et al., 2015). As focal adhesion-associated protein levels were increased or decreased by W-BR or E-BR, respectively, we examined the changes in adhesion to the ECM and migration ability of W-BR- and E-BR-treated A549 cells. Pre-treatment of A549 cells with W-BR slightly increased cell attachment to collagen I, collagen IV, and poly-d-lysine/laminin. Moreover, pre-treatment with E-BR reduced the attachment of A549 cells to the ECM in a dose-dependent manner (Figure 3D). In a transwell migration assay, serum-induced migration activity was significantly increased by W-BR to approximately 3-fold at 200 μg/ml compared with that of untreated control cells, whereas it decreased upon E-BR treatment at 100 μg/ml (Figure 3E). To further examine the migration ability of A549 cells, we performed a wound-healing assay. Untreated control A549 cells (0 μg/ml) migrated across the wound area, leading to approximately 30% and 45% healing at 24 and 48 h, respectively (Figure 3F). W-BR treatment increased wound migration compared with that of untreated control cells in a dose-dependent manner, resulting in wound healing of approximately 46.3% and 77.3% at 200 μg/ml after 24 and 48 h, respectively. In contrast, E-BR treatment significantly reduced wound migration. These results indicate that E-BR exhibits potent anti-cancer activities and is consistent with the predictions of the transcriptome analysis.

### W-BR increases HaCaT cell adhesion to collagen I, focal adhesion-associated protein levels, and migration

**FIGURE 4:**
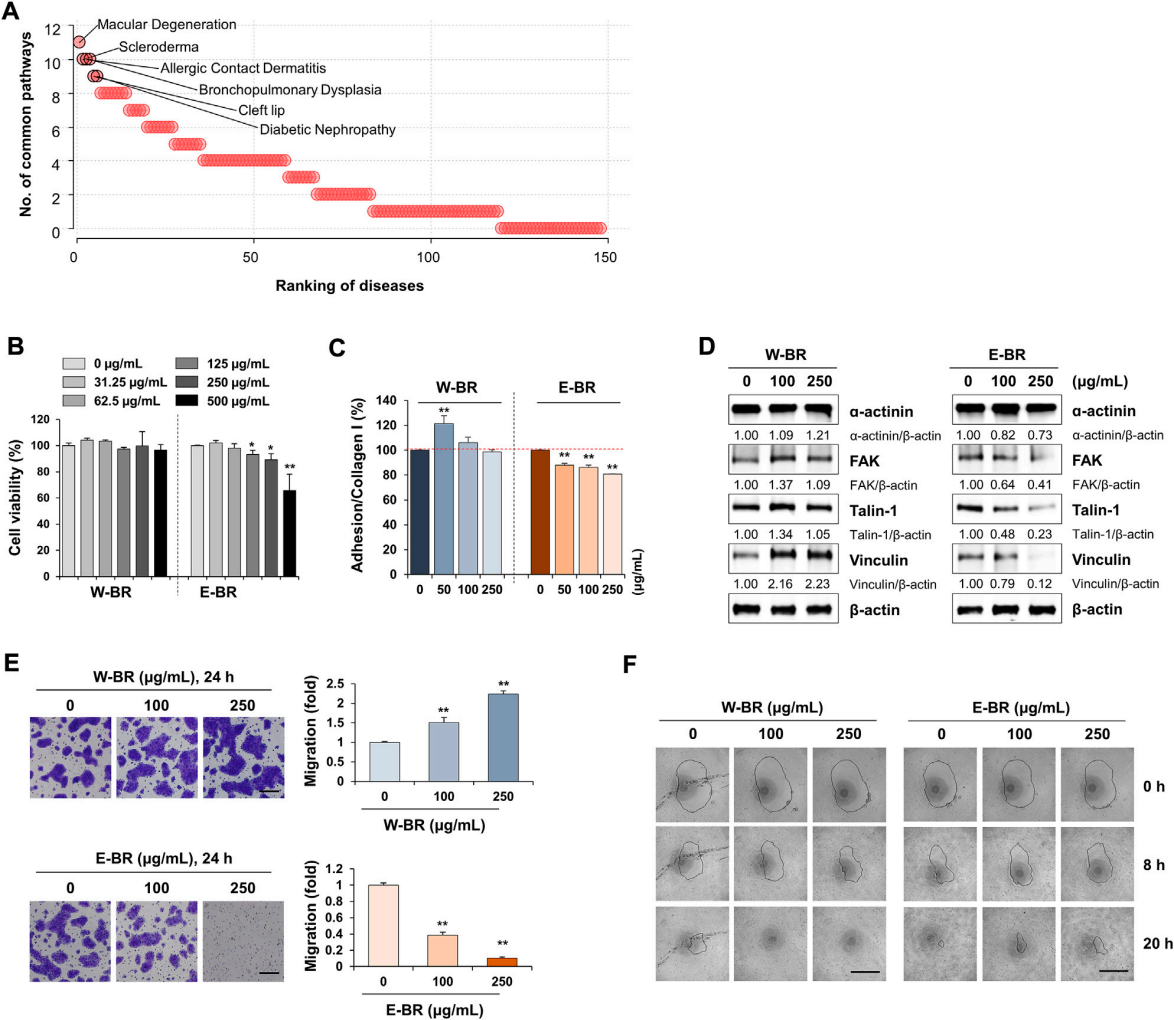
Effects of bupleuri radix water extract (W-BR) and bupleuri radix ethanol extract (E-BR) on proliferation, adhesion, and migration of HaCaT cells. **(A)** Prediction of indications of W-BR. The ranking of diseases associated with W-BR was measured as the number of common pathways that were significantly enriched in expression signatures of disease and W-BR. **(B)** HaCaT cells were treated with the indicated concentrations of W-BR or E-BR for 24 h, and then cell viability was measured. **(C)** HaCaT cells treated with or without W-BR and E-BR were added onto collagen I-coated wells. After incubating cells for 30 min, attached cells were quantitated. **(D)** The levels of ɑ-actinin, focal adhesion kinase, talin-1, and vinculin were detected by immunoblotting in W-BR or E-BR-treated HaCaT cells. Relative band intensities were calculated after normalization to β-actin expression. Data are representative of two independent experiments. **(E)** HaCaT cells were pretreated with W-BR or E-BR for 24 h and evaluated for migration ability across the transwell membrane. Scale bar = 200 μm. Original magnification × 100. **(F)** Wound healing in W-BR or E-BR-treated HaCaT cells was detected using the RadiusTM 96-well Cell Migration Assay kit. Images were taken at the indicated timepoints, and the wound area is indicated with a solid line. Data are expressed as means ± standard deviation from triplicate samples. *p < 0.05 and **p < 0.01 vs. vehicle-treated control. E-BR: bupleuri radix ethanol extract; W-BR: Bupleuri radix water extract, FAK: focal adhesion kinase.

To identify indications for W-BR, we compared the expression signatures of W-BR with those of a disease database from CREEDS (Wang et al., 2018), which provides manually curated sets of disease-related genes. Diseases were ranked by the number of the key pathways altered in opposite directions by BR treatment and the disease; The top five diseases included macular degeneration, scleroderma, allergic contact dermatitis, bronchopulmonary dysplasia, and cleft lip (Figure 4A). Scleroderma and allergic contact dermatitis are types of skin disorders, and bronchopulmonary dysplasia and cleft lip are membrane protein disorders associated with dysregulation of cell adhesion molecules during development (Bose et al., 2011; Paiva et al., 2019). Taken together, these data suggest that W-BR might have therapeutic potentialin skin disorders, particularly via enhancement of cellular adhesion.

To investigate the role of W-BR in skin wound healing, we tested human keratinocyte HaCaT cells. Keratinocytes are the major cellular components of the *epidermis* and play an important role in wound healing after injury. Proliferation, adhesion, and migration of keratinocytes are critical factors that contribute to efficient wound repair (Werner et al., 2007). Increasing concentrations of W-BR ranging from 31.25 to 500 μg/ml did not affect HaCaT cell proliferation (Figure 4B). E-BR at concentrations of up to 250 μg/ml exhibited no cytotoxicity. At non-cytotoxic concentrations, HaCaT cells treated with W-BR showed slightly higher adhesion activity to collagen I than those treated with E-BR (Figure 4C). No adhesion of HaCaT cells to collagen IV and poly-d-lysine/laminin was observed. Similar to the A549 cells, the levels of focal adhesion-associated proteins in HaCaT cells were increased by W-BR and decreased by E-BR (Figure 4D). In the transwell migration assay, W-BR-treated HaCaT cells showed greater migration activity than that showed by E-BR-treated cells (Figure 4E). Additionally, W-BR-treated HaCaT cells showed notable improvement in wound closure compared with that showed by the untreated control, whereas E-BR-treated cells showed lower wound closure activity (Figure 4F). These results indicate that W-BR may have wound healing promoting effects.

### SSa and SSd contribute to apoptosis induction and inhibitory effects on adhesion and migration

**FIGURE 5:**
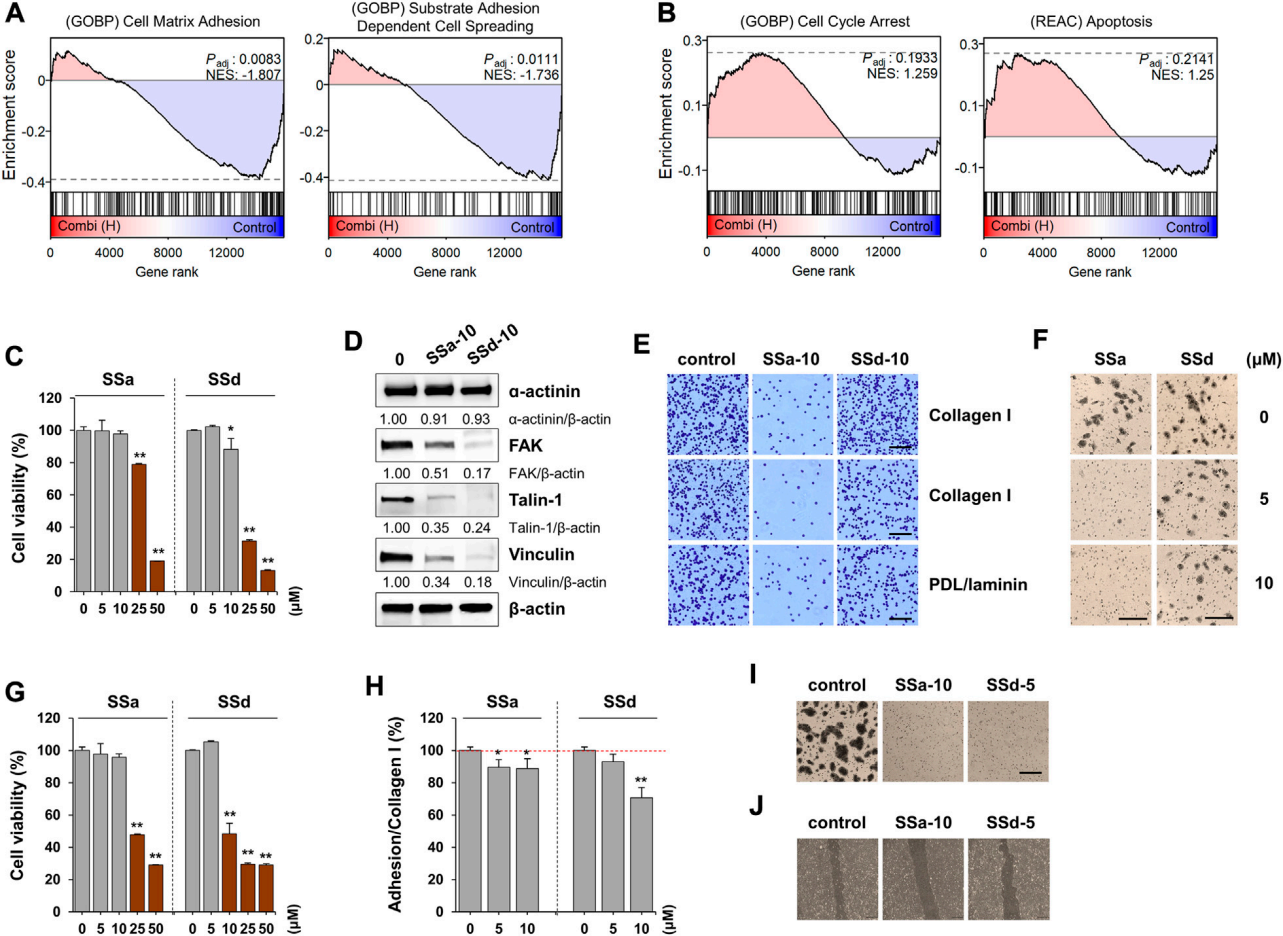
Effects of saikosaponin (SS) on proliferation, adhesion, and migration of A549 and HaCaT cells. **(A,B)** Gene set enrichment analysis results for the combination of SSs with **(A)** decreased and **(B)** increased pathways. **(C)** A549 cells seeded on 96-well culture plates were treated with SSa or SSd (0–50 μM) for 24 h. Cell viability relative to vehicle-treated cells was expressed as the mean ± SD. **(D)** A549 cells were treated with SSa (10 μM) and SSd (10 μM) for 24 h, and the protein levels of ɑ-actinin, focal adhesion kinase, talin-1, and vinculin were measured by immunoblotting. After normalization to β-actin expression, the relative band intensities were calculated. Data are representative of two independent experiments. **(E)** A549 cells were treated with SSa (10 μM) and SSd (10 μM) for 24 h. After harvesting the cells, adhesion to collagen I, collagen IV, or poly-D-Lysine/laminin was observed after crystal violet staining. Scale bar = 100 μm. Original magnification × 100. **(F)** A549 cells treated with 5 and 10 μM SSa and SSd were harvested, and their transwell migration was measured. Scale bar = 200 μm. Original magnification × 100. **(G)** HaCaT cells were treated with SSa and SSd (0–50 μM) for 24 h and the cell viability relative to vehicle-treated cells was expressed as the mean ± SD of triplicate samples. **(H)** Adhesion to collated I matrix was determined in SSa- and SSd-treated HaCaT cells, and data are expressed as the means ± SD of triplicate samples. **(I)** HaCaT cells treated with 10 μM SSa and 5 μM SSd were harvested, and their transwell migration was measured. Scale bar = 200 μm. Original magnification × 100. **(J)** Injury lines were made on HaCaT cells grown up to 90% confluence in a 12-well culture plate, and then cells were incubated with SSa (10 μM) or SSd (5 μM). After 24 h, cell migration to wound areas was monitored under an inverted microscope. *p < 0.05 and **p < 0.01 vs. vehicle-treated control.

Saikosaponins are the major components of BR extracts (Yuan et al., 2017). To verify whether these major compounds contribute to the effects elicited by E-BR or W-BR, we analyzed the transcriptome profiling from the treatment of A549 cells with a combination of the seven saikosaponins, SSa, b1–b4, c, and d. The combined effect of these seven compounds was similar to that of E-BR as the pathways of adhesion and migration decreased (Figure 5A), whereas the pathways of cell cycle arrest and apoptosis increased (Figure 5B). Considering that SSa and SSd were previously reported to inhibit cancer cells (Tsai et al., 2002; Hsu et al., 2004a) as well as adhesion and migration (Chen et al., 2013), these major compounds may be responsible for the biological effect of E-BR but not W-BR.

To identify the major active compounds contributing to the pharmacological activity of BR extracts, we first determined the cytotoxic effects of saikosaponins on A549 and HaCaT cells. SSa and SSd significantly decreased the viability of A549 and HaCaT cells, respectively, in a dose-dependent manner (Figures 5C,G). Moreover, SSb2, SSb3, SSb4, and SSc exhibited no cytotoxicity up to 50 μM, and SSb1 reduced cell viability at 50 μM (Supplementary Figure S4). As the content of SSa and SSd in E-BR was 16.52- and 12.1-fold higher, respectively, than that in W-ER, we examined whether the non-cytotoxic doses of SSa and SSd decreased the ability of adhesion and migration and reduced the levels of focal adhesion proteins. In A549 cells, SSa, and SSd suppressed the levels of FAK, talin-1, and vinculin (Figure 5D). Additionally, SSa almost completely inhibited the adhesion and migration of A549 cells, whereas SSd slightly inhibited these processes (Figures 5E,F). In HaCaT cells, adhesion to collagen I, transwell migration, and wound migration were effectively inhibited by SSa and SSd, similar to E-BR (Figures 5H–J). These results indicate that SSa and SSd contribute to the biological effect of E-BR, whereas compounds other than the examined saikosaponins mediate the wound healing effect of W-BR.

## Discussion

**FIGURE 6:**
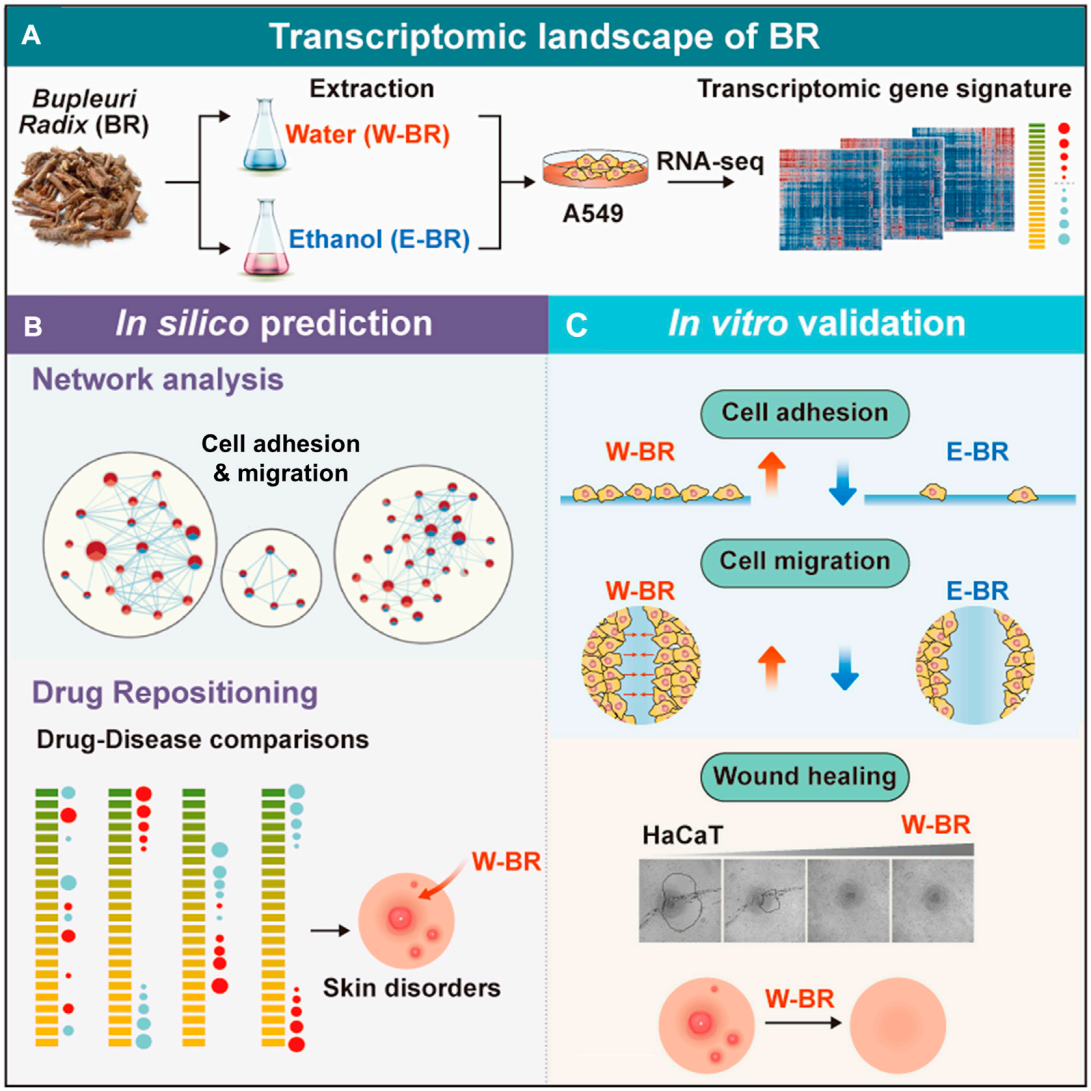
Summary of methods and findings of the study. **(A)** Transcriptome data induced by treatment of bupleuri radix (BR) extracts and their ingredients were generated using RNA-seq. **(B)** Systematic transcriptomic analysis revealed increased cell adhesion and migration in BR ethanol extract (E-BR) but not in BR water extract (W-BR). Further drug-disease comparison suggested indications for skin disorders. **(C)** Molecular experiment validated the identified molecular mechanisms of W-BR and E-BR. The wound healing effect of W-BR was confirmed in skin cells. GOBP: Gene Ontology Biological Processes; REAC: Reactome; SS: saikosponin.

To elucidate the molecular mechanism and indications of BR–a widely used herbal medicine, we adopted a systematic transcriptomics approach to analyze gene expression changes induced by treatment with BR extracts and their major components (Figure 6A). Pathway enrichment analysis supported the previously reported anti-cancer effects of BR extracts (Cheng et al., 2003; Hsu et al., 2004b). Additionally, we identified a stimulatory effect of W-BR on cell adhesion and migration as opposed to the effects of E-BR (Figure 6B). Moreover, the effects on adhesion and migration were experimentally verified in both A549 and HaCaT cells, which are normal keratinocytes derived from skin tissue (Figure 6C). The indications for wound healing of BR have been recognized not only in the East but also in the West; however, experimental studies on the molecular mechanisms of wound healing by BR have been limited (Ashour and Wink, 2011; Prieto et al., 2012). We analytically and experimentally determined that W-BR exhibits wound healing effects particularly via the activation of adhesion- and integrin-associated pathways, including FAK regulation.

Several herbal medicine formulas containing BR, such as Soshiho-tang and Shihocheongan-san, have previously been studied for treating atopic dermatitis. From *in vivo* experiments, Lee et al. suggested the therapeutic potential of Soshiho-tang, which is composed of seven herbs, on skin lesions by alleviating atopic symptoms (Lee et al., 2019). Choi et al. (2011) demonstrated the efficacy of Shihocheongan-san, which is composed of nine herbs, for atopic dermatitis based on scoring the atopic dermatitis index and eczema area and severity index. However, to the best of our knowledge, studies on BR alone on wound-healing mechanisms in the skin have not been conducted. We suggest for the first time that BR, especially W-BR, contributes to wound healing by increasing cell adhesion and migration.

Our transcriptomic analysis identified distinct mechanisms of action of ethanol and water extract of BR, E-BR, and W-BR. Previous studies have reported that ethanol extracts generally exhibit higher physiological activity than that exhibited by water extracts of herbal medicines. Lee et al. (Lee and Kim, 2020) reported that the anti-inflammatory efficiency of ethanol extract of Jakyakgamcho-Tang was 30% higher than that of its water extract. Bae et al. (2012) showed that ethanol extracts of *Rumex acetosa* provide better protection against gastric ulcers than that provided by its water extracts. Sun et al. (2007) compared ethanol extracts and water extracts of 15 herbal medicines and demonstrated that the anti-cancer effect of ethanol extracts is superior to that of water extracts. Accordingly, our study showed that E-BR showed a better anti-cancer effect than that showed by W-BR, whereas the wound-healing effect of W-BR was superior to that of E-BR. However, the mechanism underlying the differences in efficacy between ethanol and water extracts requires further investigation.

The conventional network pharmacological approach used to elucidate the molecular mechanism of herbal medicines involves the analysis of target genes and components of the herbs without considering the content of the compounds (Li and Zhang, 2013; Park et al., 2022). However, when herbs are extracted into different solvents, the contents (including the type) of the extract differ. In fact, a different composition, especially in terms of SSa and SSd, between E-BR and W-BR was detected (Choi et al., 2021). To identify the compounds contributing to the newly discovered effect of BR on cell adhesion and migration, we analyzed and performed experiments with seven major BR components, SSa, b1–4, c, and d. However, the effect of W-BR was not conclusively attributed to these major compounds, which is a limitation of this study. Although these saikosaponins are the index compounds of BR and are reported as active components of BR, their total content is lower than 10% (Choi et al., 2021). Additionally, a variety of chemical compounds have been isolated from BR, including saponins, polyacetylenes, flavonoids, lignans, fatty acids, sterols, and volatile oils (Yang et al., 2017). Further studies aimed to identify a compound or set of compounds contributing to the effect of W-BR on cell adhesion and migration may help clarify the indications of BR and develop effective naturally-derived therapies for wound healing. In addition, the indications of W-BR and compounds should be verified in an *in vivo* animal model in a follow-up study.

Our findings from transcriptome-based pathway analyses and *in vitro* validation showed how different extracts generated different biological and molecular effects, as W-BR exhibited a distinct wound-healing effect, which was absent in E-BR treatment. Transcriptomic signatures of herbal medicines after treatment with extracts can reflect the effects on the perturbed signaling pathways *via* the combinatorial action of extracted compounds. Thus, transcriptome studies can provide a molecular mechanism for finding the novel indications of herbal medicines.

## Data Availability

The datasets presented in this study can be found in online repositories. The names of the repository/repositories and accession number(s) can be found below: NCBI GEO, GSE182007.

## Abbreviations

BR, Bupleuri Radix; CMap, Connectivity Map; DMSO, dimethyl sulfoxide; GSEA, gene set enrichment analysis; E-BR, Bupleuri Radix ethanol extract; ECM, extracellular matrix; FAK, focal adhesion kinase; MoA, mode of action; SSa, saikosaponin a; SSd, saikosaponin d; W-BR, Bupleuri Radix water extract.
