# Supplementary material for: Systematic transcriptome analysis reveals molecular mechanisms and indications of bupleuri radix
Source: Front Pharmacol. 2022 Oct 11;13:1010520. doi: 10.3389/fphar.2022.1010520 (PMC9592978; doi:10.3389/fphar.2022.1010520)
Supplement: Supplementary file 1 [file DataSheet1.docx]

Supplementary Material

# Supplementary Figures

**
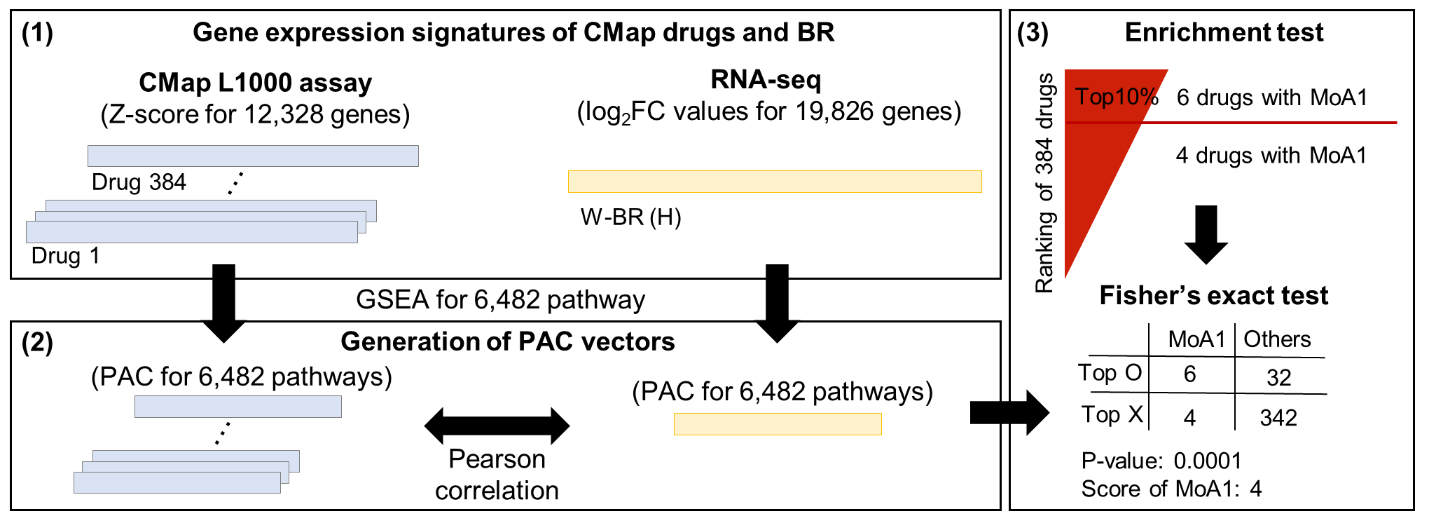
**

**Supplementary Figure 1. Schematic workflow of CMap analysis for inferring MoAs and indications of BR based on similarity of gene signatures.** This schematic illustrates an example of a procedure of scoring the association of W-BR (H) with MoA1.

**
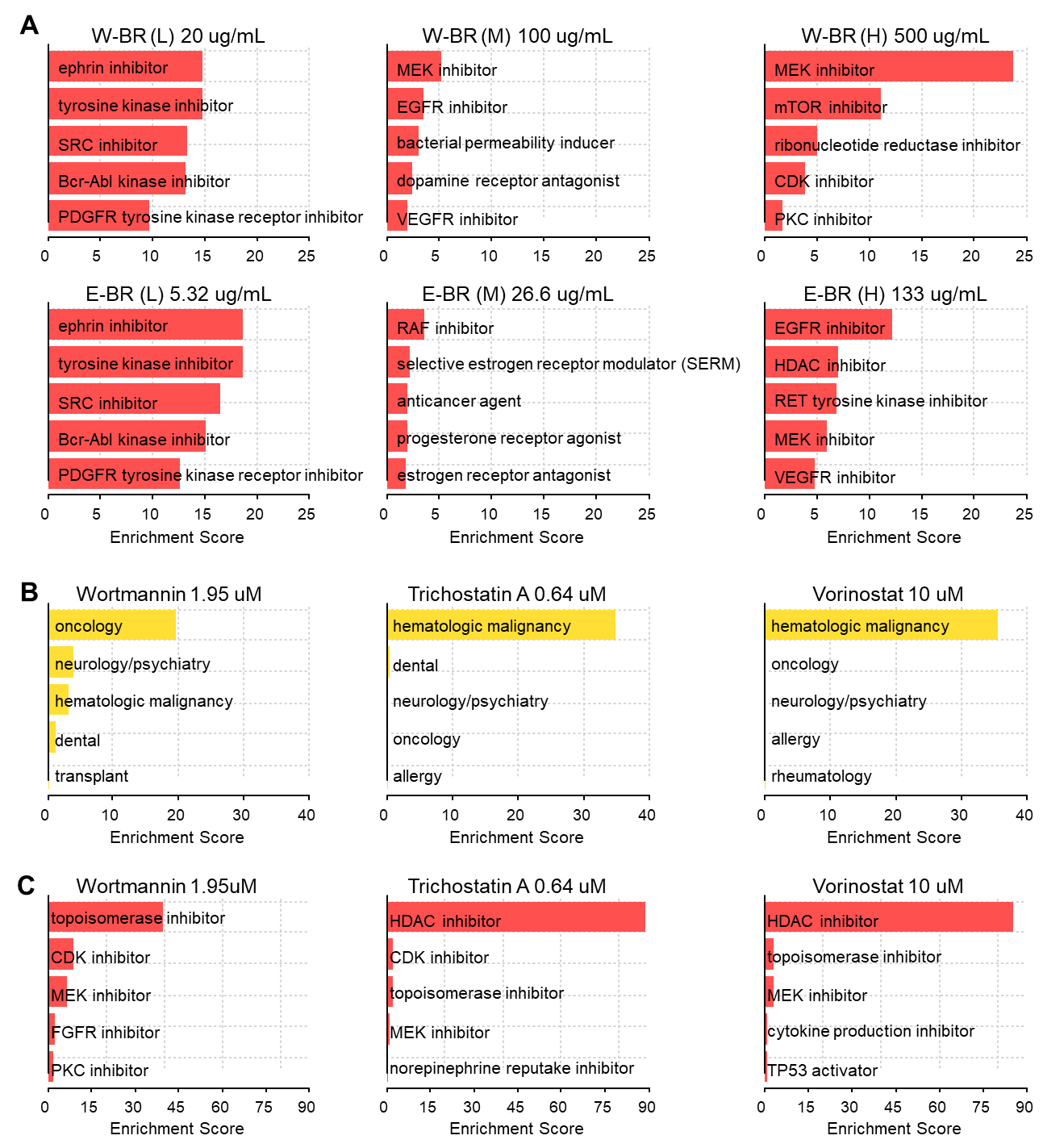
**

**Supplementary Figure 2.** **Transcriptomic signatures for W-BR, E-BR, and three positive control drugs.** (**a**) Top-enriched mode of action (MoA) classes of drugs for which expression signatures are similar to those of W-BR (upper) and E-BR (lower). (**b**) Top-enriched therapeutic areas of drugs for which expression signatures are similar to those of wortmannin, trichostatin A, and vorinostat. (**c**) Top-enriched MoA classes of drugs for which expression signatures are similar to those of wortmannin, trichostatin A, and vorinostat. Enrichment score is defined as -log10 (p-value) as estimated in the hypergeometric test.


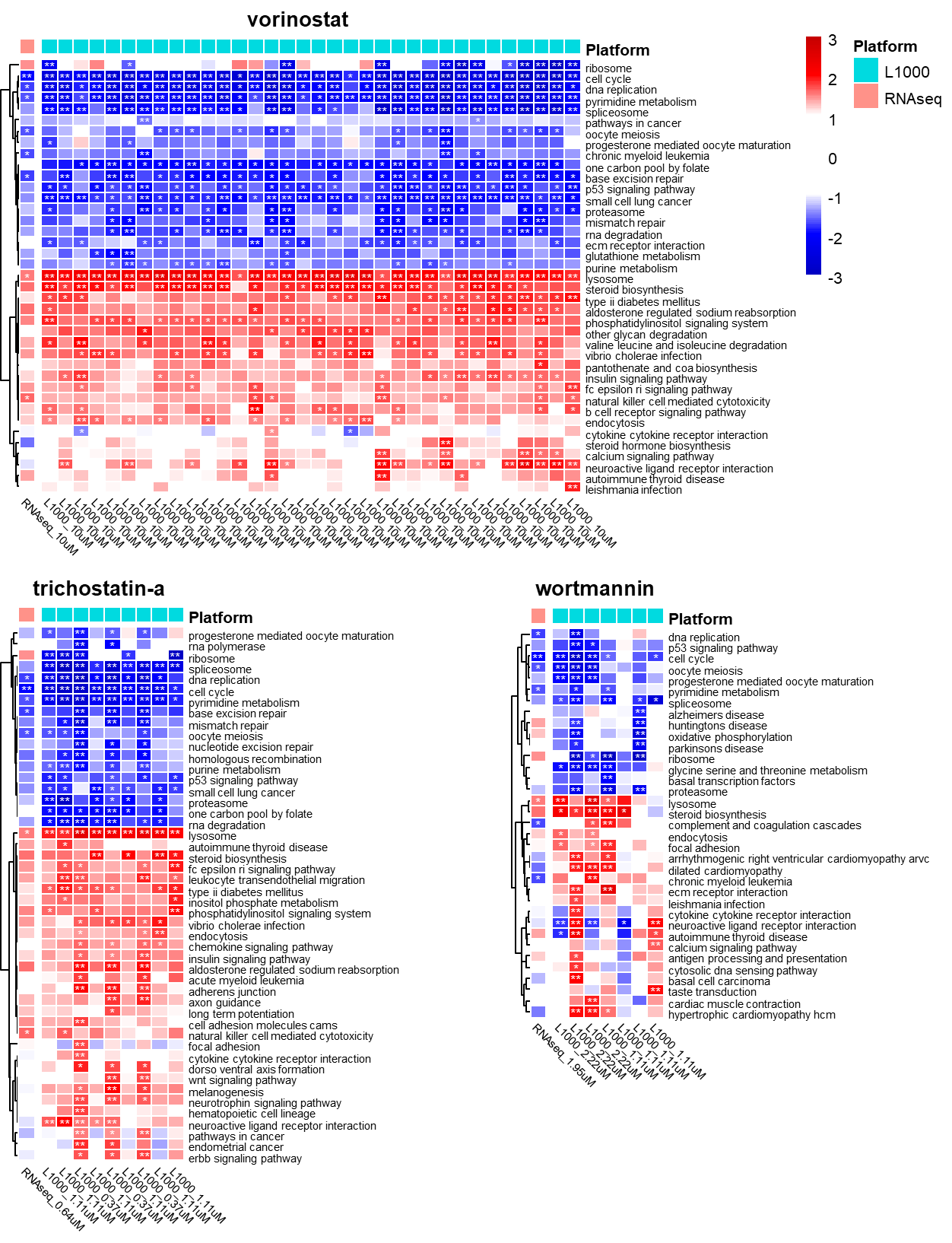


**Supplementary Figure 3.** **Altered biological pathways in A549 cells upon drug perturbation using vorinostat, trichostatin A, and wortmannin.** Color on the heatmap represents the normalized enrichment score computed by Gene Set Enrichment Analysis using KEGG pathways.


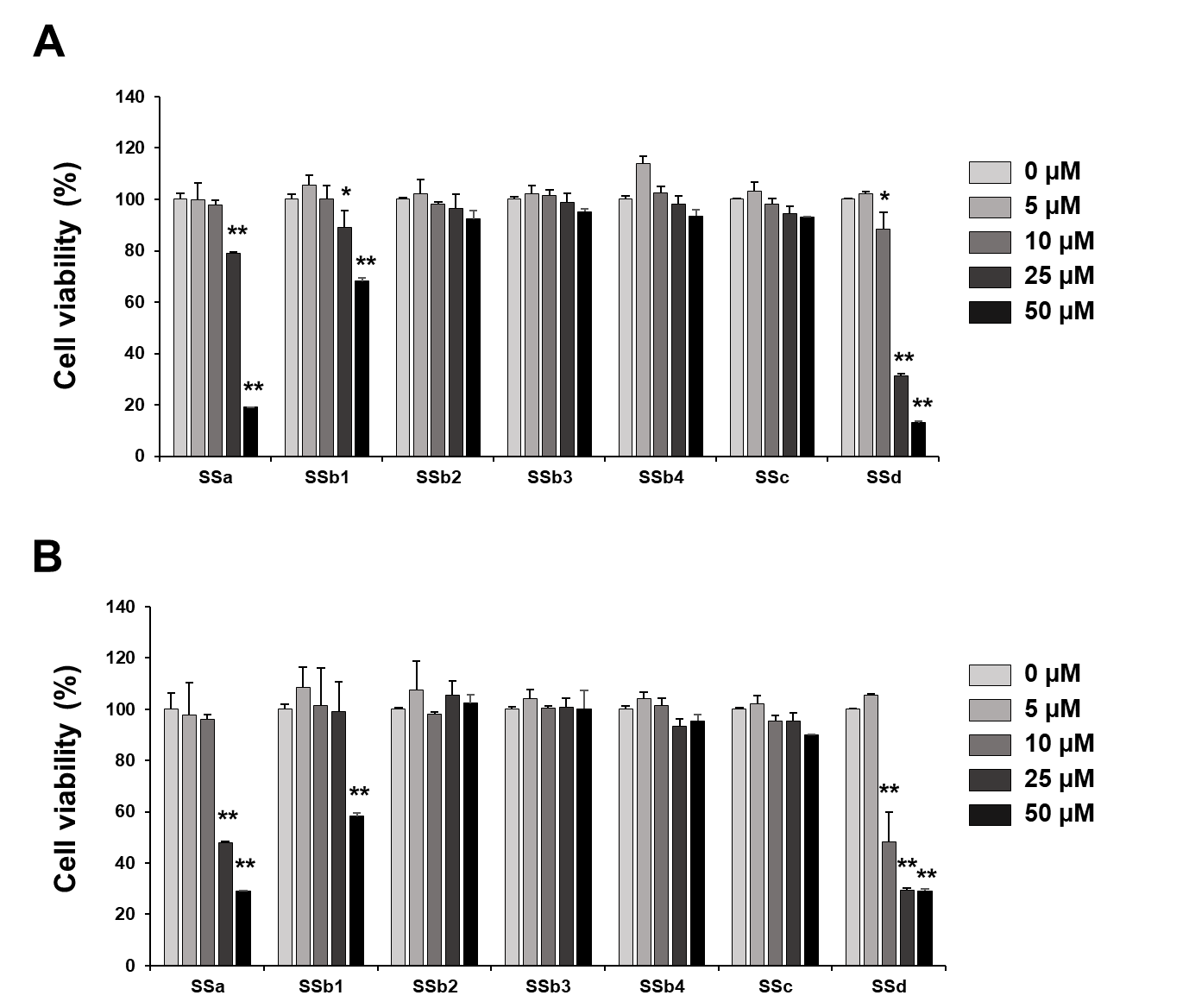


**Supplementary Figure 4.** **Cell viability assay results obtained using main compounds of BR.** A549 (**a**) and HaCaT cells (**b**) were seeded on 96-well culture plates in triplicate and treated with increasing concentrations of each saikosaponin (0–50 μM): SSa, b1-4, c, and d. After 24 h, cell viability compared with vehicle-treated cells was calculated and is expressed as mean ± standard deviation. *p < 0.05 and **p < 0.01 vs. vehicle-treated control.


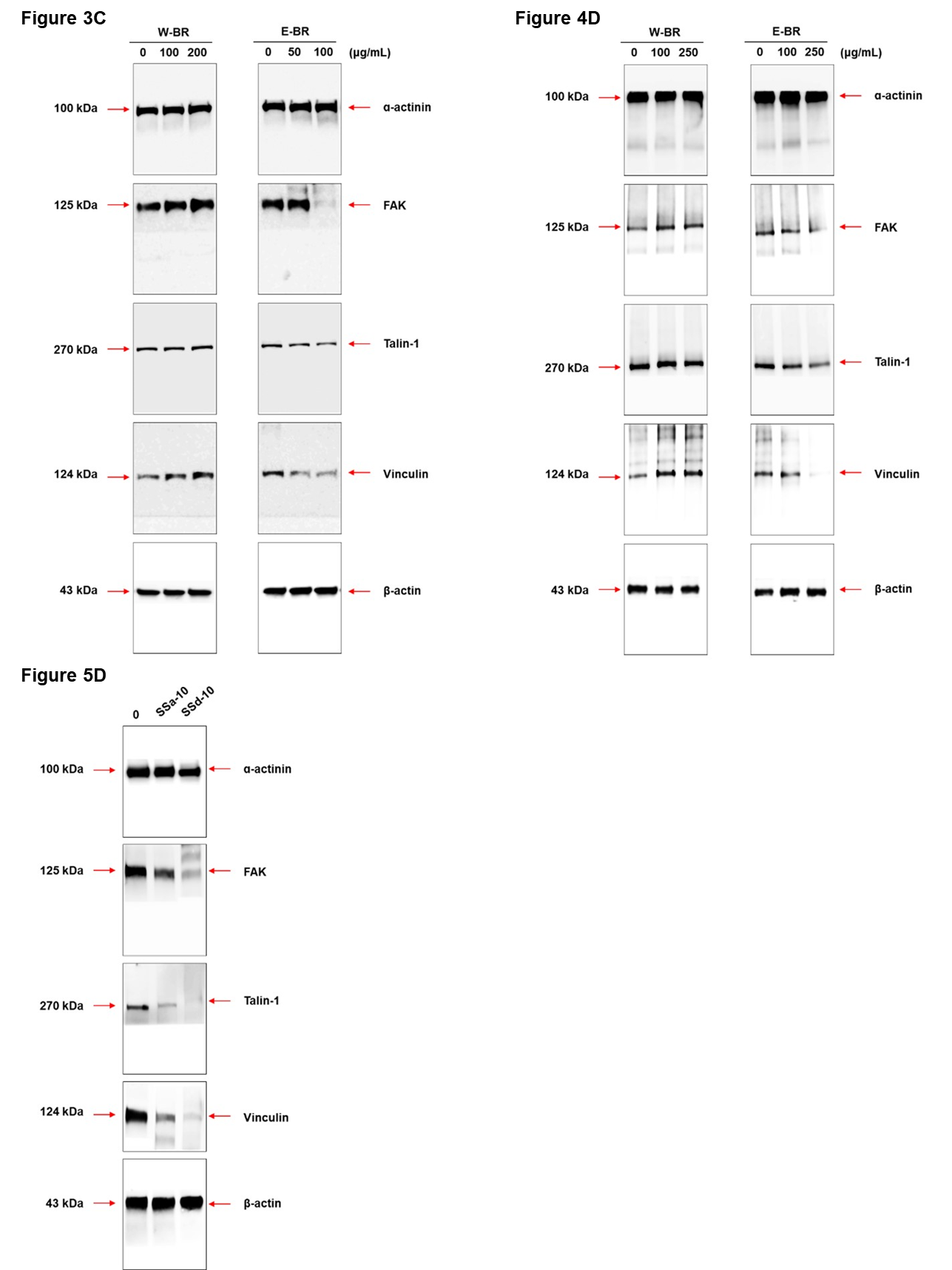


**Supplementary Figure 4.** **Whole un-cropped images of the original western blots of Figure 3C, 4D, and 5D.**
